# A Comparison Between Neoadjuvant Chemotherapy and Neoadjuvant Chemoradiotherapy in Treating Esophageal Carcinoma: A Study at a Tertiary Care Cancer Center in Suburban India

**DOI:** 10.7759/cureus.26674

**Published:** 2022-07-09

**Authors:** Balaji K Shewalkar, Ajay K Boralkar, Aditi Kaldate, Meghana Shewalkar

**Affiliations:** 1 Radiation Oncology, Government Medical College and Cancer Hospital, Aurangabad, IND; 2 Surgical Oncology, Government Medical College and Cancer Hospital, Aurangabad, IND; 3 Onco-Nutrition, Private Practice, Aurangabad, IND

**Keywords:** tumour regression, pathological response rate, neoadjuvant chemotherapy, neoadjuvant chemo radiation, ec- esophageal cancer

## Abstract

Background and objective

Esophageal carcinoma remains a disease associated with high mortality rates among patients even after receiving treatment. Management with surgery alone offers a five-year survival of only 20%. Hence adjuvant and neoadjuvant therapies were instituted to treat this condition along with surgery. Neoadjuvant chemoradiotherapy (NACRT) followed by surgery is currently the standard of care. Neoadjuvant chemotherapy (NACT) is also recommended by some authors as a method of adequate care. There is a scarcity of studies in the literature comparing NACRT with NACT. In light of this, we employed the criteria of pathological response as a primary endpoint to compare the effectiveness of NACT and NACRT in treating esophageal carcinoma.

Materials and methods

A total of 50 patients with esophageal cancer having Eastern Cooperative Oncology Group (ECOG) scores 0-2 with cancer stages cT2-T4a, cN0-N1, and cM0 were enrolled. The patients were further classified into two groups of 25 each. While one group received chemotherapy using inj. paclitaxel and carboplatin (NACT group), the other was managed with inj paclitaxel and carboplatin as well as 42 Gy of fractionated irradiation (NACRT group). Six weeks after the last dose of radiation or three weeks after chemotherapy, they were evaluated and offered transthoracic esophagectomy (TTE).

Results

Squamous cell carcinoma was found in 39 (78%) cases and 11 (22%) cases had adenocarcinoma. Pathologically complete or near-complete responses were seen in 42% of patients in the NACRT group and 22% in the NACT group.

Conclusion

While NACT and NACRT are both effective therapies for esophageal cancers, NACRT offers better tumor regression compared to NACT. Given the higher rates of complete or near-complete response in the NACRT group, NACRT is likely to offer higher overall survival rates than NACT.

## Introduction

Carcinoma of the esophagus is the eighth most common malignancy and the sixth most common cause of cancer-related mortality worldwide [1]. The prognosis in terms of the overall five-year survival rate among esophageal carcinoma patients is 15-25% [2]. When provided as a single modality, the treatment options for esophageal cancer, i.e., surgery, chemotherapy, and radiotherapy, have a poor overall survival rate and a high relapse rate. Hence, multimodality treatment protocols have gained wide acceptance and have been increasingly recommended as the standard of care therapy in these patients. Shah et al. reviewed neoadjuvant therapies in the management of esophageal cancers. They concluded that neoadjuvant chemoradiotherapy (NACRT) has the best complete pathologic response rates and improves five-year survival rates in locally advanced esophageal cancer patients. They also highlighted the paucity of studies in the literature in terms of comparing outcomes related to various multimodality treatment options. It was suggested that more studies should be conducted to compare the effectiveness of NACRT with other treatment options [3]. Tumour regression grade assessed by the Ryan score is an effective system for the evaluation of the outcomes of neoadjuvant therapy. The Ryan score has helped predict the risk of lymph node or distant metastasis, as well as disease-free survival and overall survival. The approach outlined by Ryan et al. among pathologists has been incorporated into the College of American Pathologists templates [4]. The present study compares NACRT with neoadjuvant chemotherapy (NACT) in the treatment of esophageal carcinoma based on the modified Ryan scheme for tumor regression score.

## Materials and methods

This study was conducted at the Department of Radiotherapy, Oncology, and Surgical Oncology of the State Cancer Institute. After obtaining approval from the Institutional Ethics Committee vide letter number Pharma/IEC-GMCA/524/2019 dated 17 October 2019, 50 patients with esophageal cancer with Eastern Cooperative Oncology Group (ECOG) scores [5] 0-2 and cancer stages [6] cT2-T4a, cN0-N1, and cM0 were selected. Patients with cervical esophageal cancers, those with an ECOG score ≥3, patients with previous radiation treatment, and pregnant and lactating patients were excluded from the study. The patients were further classified into two groups of 25 each: the NACT group and the NACRT group. Detailed history and the results of physical examination, blood biochemistry, upper gastrointestinal (GI) endoscopy, biopsy, and contrast-enhanced CT (CECT) of the neck, chest, abdomen, and pulmonary function tests were obtained.

All patients with locally advanced esophageal cancer with ECOG scores of 0-2 were registered and a detailed clinical history was taken via a thorough clinical examination. The patients were further investigated with routine blood investigations and special investigations; TNM staging was done with CECT thorax and upper abdomen before initiating NACT or NACRT. Those patients who met the criteria for surgery underwent surgery, i.e., transthoracic esophagectomy (TTE).

Neoadjuvant chemotherapy group

The patients in this group received inj. paclitaxel 80 mg/m^2^ IV plus inj. carboplatin AUC2 IV weekly for six cycles. They underwent assessment using CECT thorax and upper abdomen three weeks after the last dose of chemotherapy followed by surgical evaluation.

Neoadjuvant chemoradiotherapy group

The patients in this group underwent the following modalities of treatment:

• 42 Gy of fractionated irradiation given in 20 fractions, five fractions per week over four weeks with three-dimensional conformal radiotherapy/intensity-modulated radiotherapy (3DCRT/IMRT).

• Concurrent chemotherapy involving inj. paclitaxel 60 mg/m^2^ IV plus inj. carboplatin AUC2 IV weekly x six cycles. Radiation with 3DCRT/IMRT was followed by assessment using CECT thorax and upper abdomen six weeks after the last fraction of radiotherapy, which was followed by surgical evaluation.

Complete blood biochemistry and complete blood count (CBC) were obtained before, during, and after chemotherapy. Chemotherapy-related toxicities were monitored on a weekly basis, and dose modifications were allowed according to patients’ general conditions. The patients were evaluated with a CECT scan three weeks after the last dose of chemotherapy. Afterward, the patients were evaluated for surgery and then underwent TTE. In the NACRT group, 42-Gy radiation was provided in 20 fractions as five fractions per week with 3DCRT or IMRT. Chemotherapy was concurrently given with paclitaxel 60 mg/m^2^ and carboplatin AUC2. The response was assessed through a CECT scan six weeks after the last fraction of radiotherapy. This was followed by surgical evaluation and TTE.

Radiotherapy details

After pre-treatment with CT-based simulations with oral contrast and 5-mm sections, the relevant data were obtained. The transfer to the planning system and contouring was done and the plan was approved by a radiation oncologist. Radiation was delivered with 6 and 10 MV photons through 3DCRT/IMRT. A dose of 2.1 Gy per fraction was employed to cover the planned target volume by using the Elekta linear accelerator (Synergy, Elekta, Stockholm, Sweden). The gross tumor volume was contoured through GI endoscopy and CT scan. The clinical target volume included a 1-cm expansion of the gross target volume circumferentially and a 5-cm extension of the cephalad and caudad regions along with lymph nodes in the mediastinum and supraclavicular area for mid-thoracic tumors and coeliac nodes for lower thoracic and gastroesophageal junction tumors. Dose constraints for all organs at risk (OARs) such as the lungs (<20 Gy), heart (<30% V[E3] 30 Gy), and spinal cord (<45 Gy) were calculated.

Surgery details

Of the 50 patients, 15 patients did not undergo esophagectomy, five were deemed unfit for surgery, and seven defaulted the treatment or refused surgery. Three patients in the NACRT arm died before the completion of the six-week waiting period after the last radiation dose. The patients were evaluated for anesthesia administration and then provided chest physiotherapy, hydration, and nutritional treatment, as advised by an onco-nutritionist. Patients were then subjected to TTE. Esophagectomy was performed with a good circumferential margin. Periesophageal, paraaortic, subcarinal, retrotracheal, recurrent laryngeal nerve, and aortopulmonary nodes were excised. Furthermore, abdominal celiac nodes were excised. Additionally, in the neck region, supraclavicular nodes along with lower jugular nodes and cervical recurrent laryngeal nerve nodes were excised. Patients were electively ventilated for 12-24 hours. Enteral feeds were initiated from day one postoperatively and oral feed was administered by day five or six.

The pathological assessment was performed based on the modified Ryan scheme for tumor regression score. The Common Terminology Criteria for Adverse Events (CTCAE) Version 5.0 were used for reporting toxicity.

## Results

The majority of the patients were in the age group of 51-60 years, i.e., 11 (44%) patients in the NACT group and 12 (48%) in the NACRT group. The mean age of the patients was 54.7 ± 9.4 years (range: 31-70 years) in the NACT group as compared to 53.3 ± 8.2 years (range: 40-70 years) in the NACRT group. A male predominance was noted in both groups, with 15 (60%) males and 10 (40%) females in both. The male-to-female ratio was 1.5:1.

The location of the tumor was middle one-third in 23 patients, and in 27, it was lower third including esophagogastric junction tumors. Out of the total 50 cases, 17 had exophytic growth (proliferative and polypoidal) and 33 had endophytic (ulcerative and stricturous) growth.

The majority of the patients in the study had squamous cell carcinoma: 20 (80%) patients in NACT and 19 (76%) in the NACRT group. Adenocarcinoma was seen in five (20%) and six (24%) patients respectively. Nearly three-quarters of patients (72%) presented with advanced-stage disease. They were either T3 or T4 in their tumor stage or had lymph nodes or a combination of both. The randomized allocation to two neoadjuvant protocols yielded similar outcomes in terms of the stage (Table 1).

**Table 1:** TNM staging as per the Eighth edition of the American Joint Committee on Cancer staging T category describes the primary tumor site and size; N category describes the regional lymph node involvement; M category describes the presence or otherwise of distant metastatic spread NACT: neoadjuvant chemotherapy; NACRT: neoadjuvant chemoradiotherapy

Stage	NACT, n (%)	NACRT, n (%)
cT2N0M0	8 (32%)	6 (24%)
cT2N1M0	4 (16%)	2 (8%)
cT3N0M0	2 (8%)	4 (16%)
cT3N+M0	7 (28%)	10 (40%)
cT4N+M0	4 (16%)	3 (12%)
Total	25 (100%)	25 (100%)

Eighteen patients underwent surgery in the NACT group and 17 in the NACRT group. Three patients in the NACT group and two in the NACRT group were unfit for surgery. Four patients in the NACT and three patients in the NACRT group defaulted in treatment. After the completion of the NACRT protocol, there were three fatalities.

Postoperatively, the complete or near-complete response was observed at a higher rate in the NACRT group: 42% vs. 22% in the NACT group (Table 2).

**Table 2:** Comparision of pathological responses between the two groups (modified Ryan scheme for tumor regression score) NACT: neoadjuvant chemotherapy; NACRT: neoadjuvant chemoradiotherapy

Response	Tumor regression score	Groups	
		NACT (n=18), n (%)	NACRT (n=17), n (%)
Complete	0	2 (11.1%)	4 (23.5%)
Near-complete	1	2 (11.1%)	3 (17.6%)
Partial response	2	10 (55.5%)	8 (47%)
Poor or no response	3	4 (22.2%)	2 (11.7%)

According to the CTCAE criteria for hematological toxicity (leucopenia), in the NACT group, grade III toxicity was seen in one patient at week one, in three patients at week two, two patients at week three, three patients at week four, two patients at week five, and one patient at week six. Grade IV toxicity was seen in one patient at week four. Meanwhile, in the NACRT group, grade III toxicity was seen in one patient at week two, five patients at week three, two patients at week four, two patients at week five, and three patients at week six. Grade IV toxicity was seen in two patients at week five.

As per the CTCAE criteria for hematological toxicity (thrombocytopenia), in the NACT group, grade III toxicity was seen in three patients at week two, one patient at week four, and three patients at week five. Grade IV toxicity was seen in one patient at week four. In the NACRT group, grade III toxicity was seen in one patient at week two, one patient at week four, and five patients at week five. Grade IV toxicity was not observed in this group.

According to the CTCAE criteria for gastrointestinal toxicity (nausea), in the NACT group, grade III toxicity was seen in two patients at week two, three patients at week three, five patients at week four, two patients at week five, and four patients at week six. Grade IV toxicity was seen in one patient at week five. In the NACRT group, grade III toxicity was seen in one patient at week two, four patients at week three, and six patients each at week four, week five, and week six. Grade IV toxicity was seen in one patient each at week five and week six.

According to the CTCAE criteria for gastrointestinal toxicity (vomiting), in the NACT group, grade III toxicity was seen in two patients at week four, four patients at week five, and three patients at week six. Grade IV toxicity was not seen in this group. In the NACRT group, grade III toxicity was seen in two patients each at week one and week two, three patients at week three, five patients at week four, seven patients at week five, and six patients at week six. Grade IV toxicity was not seen in this group.

According to the CTCAE criteria for gastrointestinal toxicity (esophagitis), in the NACT group, grade III toxicity was seen in one patient each at week four, week five, and week six. Grade IV toxicity was not seen in this group. In the NACRT group, grade III toxicity was seen in one patient each at week one and week two, three patients at week three, four patients at week four, and three patients each at week five and week six. Grade IV toxicity was not seen in this group.

## Discussion

The complete or near-complete response was observed post-surgery at a higher rate in the NACRT group: 42% vs 22% in the NACT group.

Von Döbeln et al. [7] conducted a phase II randomized trial that compared NACRT with the NACT in treating resectable esophageal cancer, involving 181 patients from Sweden and Norway. The early results were similar to our findings. The authors reported that the NACRT group had good outcomes regarding the histopathological response in the primary tumor (28% vs. 9% in the NACT group). The five-year disease progression-free survival in the NACRT group was 38.9% while it was 33% in the NACT group. The five-year overall survival was 42.2% in the NACRT group and 39.6% in the NACT group. The complications observed with treatment were similar in both groups. The authors concluded that although there was a better tumor tissue response in the NACRT group, the advantages in terms of survival were insignificant.

Li et al. [8] recruited 170 patients with locally advanced adenocarcinoma of the esophagogastric junction. They were treated with neoadjuvant chemotherapy with or without concurrent radiotherapy at the Hebei Medical University. The median follow-up was 41.2 months for surviving patients. The three-year overall survival rate was 55% in the NACRT group and 38.3% in the NACT group. The pathological complete response rate was 17% in the NACRT group and 1.9% in the NACT group. There was no significant difference in postoperative complications between the groups. The authors suggested that NACRT had better survival and complete pathological response rates than NACT in locally advanced cases of adenocarcinoma of the esophagogastric junction.

Similarly, Morgan et al. have reported that NACRT is a treatment modality with satisfactory outcomes [9]. Zhao et al. [10] conducted a meta-analysis to compare the efficacy between NACRT and NACT. They observed that NACRT is a treatment option with the significant benefits of long-term survival in esophageal and gastroesophageal cancer patients. Also, the meta-analysis by Deng et al. [11] found that preoperative NACRT was a better option than NACT for treating locally advanced esophageal squamous cell carcinoma. Pasquali et al. conducted a network meta-analysis [12], which added to the evidence endorsing the influential role of NACRT over NACT in the management of both squamous cell carcinoma and adenocarcinoma of the esophagus. However, research studies have documented varying observations while comparing NACRT with NACT followed by surgery and surgery alone [13-16].

This study has a few limitations. Primarily, this was a single-center study with a small sample size. Also, there was no long-term follow-up of patients. Further studies addressing these limitations are required among the population from our region to better understand the prognosis after NACRT and NACT in esophageal cancer patients.

## Conclusions

Based on our findings, surgery alone is insufficient in the treatment of advanced esophageal tumors. Neoadjuvant therapies followed by esophagectomy are the standard treatment currently. According to pathological evaluations, NACRT is a better treatment option compared to NACT. This study had a small sample size, and hence studies with larger sample sizes are required to shed further light on the pathological responses. Furthermore, long-term follow-ups are required to better evaluate disease-free and overall survival rates. However, based on the study findings, we recommend NACRT as a more suitable neoadjuvant treatment modality for esophageal cancers than NACT.
